# Effects of CNS Demyelination and Myelin Recovery on Urinary Physiology

**DOI:** 10.1093/geroni/igaa057.393

**Published:** 2020-12-16

**Authors:** Ramalakshmi Ramasamy, Dawn Rosenberg, Cara Hardy, Stephen Crocker, Phillip Smith

**Affiliations:** 1 UConn Health, Farminton, Connecticut, United States; 2 UConn Health, Farmington, Connecticut, United States

## Abstract

Multiple sclerosis (MS) is a chronic inflammatory demyelinating disease of the central nervous system (CNS). Of note, over 80% of MS patients have urinary symptoms as one of their earliest symptoms. Since MS patients often live into older age, urinary incontinence and retention are significant problems for which few if any effective preventive or therapeutic options are available. The mechanisms by which MS contributes to urinary dysfunction are not well understood. We propose to elucidate the impact of demyelination on urinary performance using the cuprizone model, a model used to study the effects of CNS demyelination and spontaneous remyelination. We hypothesize that CNS demyelination in the cuprizone model will result in aberrant changes in urinary function, and that after remyelination occurs this dysfunction will be alleviated. C57Bl/6 mice were treated with cuprizone (0.2% w/w) for four weeks to induce demyelination. One group was allowed four additional weeks to recover from demyelination, while the other continued cuprizone treatment. Following this eight-week treatment, pressure/flow cystometry, electromyography, and molecular studies were performed to assess demyelination-induced differences in urinary performance. Cuprizone-recovery mice displayed improvements in cystometric function compared to their demyelinated littermates, as seen through improved volume sensitivity and voiding efficiency. Pharmacologic studies showed no significant changes in contractile responsiveness. Thus, we conclude that CNS demyelination results in aging-like phenotype and urinary dysfunction consistent with that observed in clinical disease. Therapeutics aimed at increasing the remyelination potential of the CNS neurons offer the possibility of alleviating urinary dysfunction associated with MS.

